# Knowledge and experience of midwives and gynecologists about manual rotation of persistent occiput posterior position

**DOI:** 10.1186/s12884-023-05797-x

**Published:** 2023-06-30

**Authors:** Pouran Allahbakhshi Nasab, Marzeyeh Loripoor, Sakineh Mirzaei Khalilabadi

**Affiliations:** 1 Department of midwifery, School of Nursing and Midwifery, Geriatric care research center, Rafsanjan University of Medical Sciences, Rafsanjan, Iran; 2 Department of obstetrics and gynecology, Rafsanjan university of medical sciences, Rafsanjan, Iran

**Keywords:** Persistent occiput posterior position, Manual rotation, Vaginal delivery, Instrumental delivery, Cesarean section

## Abstract

**Background:**

management of persistent occiput posterior position has always been controversial. Manual rotation by a delivery operator can reduce instrumental delivery and cesarean section.

**Aim:**

This study aims to determine the knowledge and experience of midwives and gynecologists about manual rotation of persistent occiput posterior position.

**Methods:**

This descriptive cross-sectional study was performed in 2022. The questionnaire link was sent to 300 participating midwives and gynecologists via WhatsApp Messenger. Two hundred sixty-two participants completed the questionnaire. Data analysis was performed using SPSS22 statistical software and descriptive statistics.

**Results:**

189 people (73.3%) had limited information about this technique, and 240 (93%) had never performed it. If this technique is recognized as a safe intervention and is included in the national protocol, 239 people (92.6%) want to learn, and 212 (82.2%) are willing to do it.

**Conclusion:**

According to the results, the knowledge and skills of midwives and gynecologists need to be trained and improved for manual rotation of persistent occiput posterior position.

**Supplementary Information:**

The online version contains supplementary material available at 10.1186/s12884-023-05797-x.

## Background

Persistent occiput posterior position occurs in 40% of deliveries. The majority spontaneously rotate to anterior positions, and 1.6-8% of cases do not rotate until delivery, called persistent occiput posterior position [1, 2]. About 33.3% of malposition and %93.5 of diastasis during labor is persistent occiput posterior position.

In women with fetal persistent occiput posterior position, interventions, maternal and neonatal complications such as prolonged labor, cesarean delivery, instrumental delivery postpartum hemorrhage, as well as the third- and fourth-degree tears increase [2]. Neonatal complications include birth trauma, low Apgar score, neonatal acidity, and the possibility of hospitalization in the NICU [3]. Further, it increases the risk of caput succedaneum, cranial hematoma, and intrauterine fetal distress [4, 5]. 70% of persistent occiput posterior position cases are eventually delivered by cesarean Section [6].

Exercises and changes in the mother’s position during labor may be recommended to correct persistent occiput posterior position; however, there is disagreement about their effectiveness [7–11].

Approaches and opinions of treatment teams about the management of the second stage of labor in the persistent occiput posterior position are different [2, 12, 13]. Management of persistent occiput posterior position depends on the experience and skill of the delivery operator and available scientific awareness [8].

Expected treatment is appropriate if the fetal heart rate and labor progression are satisfactory. However, despite proper uterine contractions and the mother’s pushing, the descent may last or stop. The appropriate treatment for prolonged or stopped second stage is not known. For this purpose, a few randomized and non-randomized trials have been performed and compared different approaches, including rotation versus expected treatment, manual/ forceps rotation, instrumental delivery, or cesarean Section [8].

Manual rotation is one of the effective methods to increase the chance of vaginal delivery in the persistent occiput posterior position [1, 2]. This simple method has been one of obstetrics and gynecology practices since the early 1930s [11].

For women with the prolonged second stage and adequate contractions and appropriate pelvis, it is recommended to try manual rotation to the anterior position, providing a high success chance (up to 93%). This may shorten the second stage of labor and increase the chance of vaginal delivery [8, 14] with a low risk for mother and baby (cervical rupture and fetal distress) [8, 15]. However, it is performed by a few obstetricians and midwives and has not been accepted by most [6, 16].

Phipps et al. (2014) showed that manual rotation of persistent occiput posterior is accepted by the majority of Australian midwives but performed by the minority. The researchers concluded that if manual rotation was recognized as a way to facilitate labor, midwives would be more proactive in introducing the procedure as a widespread clinical method [6].

Phipps et al. [17] concluded that gynecologists would perform this procedure routinely if instrumental delivery were reduced by 50% using manual rotation of persistent occiput posterior.

Due to the high success rate of manual rotation and its safety [2, 8, 18] and the lack of a similar study in Iran, this study aimed to investigate the knowledge and experience of midwives and gynecologists in performing manual rotation of persistent occiput posterior position in Iranian educational and medical centers.

## Methods

This descriptive cross-sectional study was conducted with the ethics code IR.RUMS.REC.1400.168 from Rafsanjan University of Medical Sciences in April and May 2022.

According to Phipps et al. [6], in Australia, the sample size was calculated at 250 people; However, 300 people were determined, considering the possible fall.

$$n = \frac{{{Z^2}pq}}{{{d^2}}},\ q = 1 - p$$

Inclusion criteria included a willingness to participate in the study, employment in labor and delivery, and at least one year of work experience; if the questionnaires were not completed entirely, they would be excluded from the study.

The research tool was a researcher-made questionnaire that included one part of demographic information, such as age, education level, work experience, and city name, and one other part of the topic-specific questions, including knowledge, experience, and the need to be trained in manual rotation of occiput posterior position, with yes and no answers. This questionnaire was compiled based on the review of texts and articles in this field [1, 2, 6, 16], and its validity was provided through content validity and the opinions of 10 faculty members related to the subject. In order to evaluate the reliability of Cronbach’s alpha, 0.7 was obtained through testing-retesting with the participation of 20 people with an interval of two weeks.

The questionnaire was posted on the Pors Line website, and its link was sent by the Midwifery Office of the Ministry of Health using WhatsApp Messenger in a random way for midwives working in educational and medical centers all over Iran, as well as gynecologists whose mobile phone numbers could be obtained through the association of gynecologist. The questionnaire link was sent to 300 people, 262 of whom completed it. First, the goals of the project were explained to them, and they were then asked to answer questions anonymously.

Data analysis was performed by SPSS statistical software (version 22) and using descriptive statistics (frequency distribution tables and central indicators).

## Results

Of the participants, 232 (89.9%) were midwives, and 30 (10.1%) were gynecologists. The mean age of the participants was 36.9 8 8.9 in the age range of 22–64 years; the average work experience was 11 ± 8.3 in the range of 1–44 years.

Based on the results, 189 participants (73.3%) had heard about manual rotation of the occiput posterior position, and 69 (26.7%) had not heard. Of those who had heard about this technique, 15 (7.4%) had heard from fellow doctors, 25 (12.3%) from fellow midwives, 107 (52.7%) through university education, and 56 (27.6%) from a valid reference in the field.

Sixty-eight individuals (26.4%) observed this technique, and 190 individuals (73.6%) did not. Among those who observed the technique, 23 (18.4%) observed via film, 19 (15.2%) via image, 21 (16.8%) in bed by a doctor, 18 (14.4%) in bed by midwives, and 44 (35.2%) in other ways.

About performing manual rotation of persistent occiput posterior position at the patient’s bedside, 18 people (7%) had performed this technique with a frequency of 2.3 ± 1.1 times, and 240 (93%) had not.

One hundred eighteen persons (45.7%) considered manual rotation of persistent occiput posterior as an acceptable action before instrumental delivery, 70 people (27.1%) were not sure about the technique, and 70 people (27.1%) did not know about it.

Concerning the time of manual rotation, 46 persons (17.8%) stated the first stage of labor, 157 persons (60.9%) the second stage, 4 persons (1.6%) non-importance of the stage, and 51 persons (19.8%) %) had no information about this.

One hundred twenty-three people (47.7%) considered this technique a scientific and valid intervention, 51 people (19.8%) considered it invalid, and 84 people (32.6%) did not know about it.

Two hundred twelve persons (82.2%) answered positively, and 46 persons (17.8%) answered negatively to the question “Would you like to do manual rotation if this technique reduces maternal and neonatal complications, instrumental delivery, and cesarean section, and be introduced as a safe and uncomplicated intervention?“

Fourteen individuals (5.4%) never correctly diagnosed occiput posterior position, 95 persons (36.8%) rarely, 137 persons (53%) most often, and 12 persons (4.7%) always recognized the position correctly.

About the position to help rotate the fetal head, the knee-chest position was used 66.7%, lying on the side 38%, asymmetric position 34.4%, walking 31.8%, and others were used 13.6%; 14.3% did not intervene.

Two hundred thirty-nine (92.6%) desired to learn this technique, and 19 (7.4%) did not.

According to the survey, most of the participants stated that the reason for not doing this technique was the lack of sufficient training and practical skills, as well as not mentioning it in the national protocol.

## Discussion

The results of this study showed that the majority of midwives and gynecologists had acquired only a limited theoretical knowledge of manual rotation of persistent occiput posterior technique at university and had not observed or practiced it.

Although most of them considered doing this technique before delivery as an acceptable method, they had not performed it.

Currently, the most recommended posture in persistent occiput posterior position is knee-chest position, lying on the side, and asymmetrical postures, while there is not enough evidence for their effectiveness [7–11]. While manual rotation of persistent occiput posterior position reduces childbirth, maternal, and neonatal complications, as well as the rate of cesarean Sects. [2, 8, 12, 16, 18–20], according to the results of this study, it is rarely done by gynecologists and midwives.

A study by Phipps et al. (2014) in Australia agrees with the present research, showing that manual rotation is accepted by most Australian midwives but performed by a minority. The researchers concluded that if manual rotation was recognized as a way to facilitate labor, midwives would be more likely to perform it prophylactically; they also suggested the method be introduced as a widespread clinical procedure [6].

Phipps et al. (2012), determining the performance of gynecologists in manual rotation of persistent occiput posterior in the second stage of labor consistent with our study, showed that if instrumental delivery is reduced up to 50% with this procedure, gynecologists will perform it routinely [17].

Jeffrey et al. (2021), determining whether manual rotation of persistent occiput posterior position reduces maternal and neonatal complications, showed that the length of the second stage of labor was shorter in the group of manual rotation; however, more clinical trials with more samples were suggested [13].

Lin Yang et al. ( 2018) compared the effect of maternal position, manual rotation, and instrumental rotation on persistent occiput posterior position and showed that the length of labor was shorter in the intervention group. Pain, blood loss 2 h after delivery, and episiotomy rate were significantly lower in the intervention group, and the difference was statistically significant [18].

Exercises and changes in the mother’s position during labor may be recommended to correct the persistent occiput posterior position. However, some studies have shown these items not to increase the chances of spontaneous rotation, and there is disagreement about this [7–11].

Desbriere et al. (2013) studied the effect of changes in maternal position during labor to help rotation of persistent occiput posterior and showed that this item had no effect [7].

Le Ray et al. (2016) investigated the effect of asymmetric lateral position on the rotation of the occiput posterior position, showing that this situation does not cause rotation of the occiput posterior and suggesting that other positions be considered [9].

Blank et al. (2021) aimed to determine whether prophylactic rotation of persistent occiput posterior in beginning the second stage of labor reduces the chance of instrumental delivery and showed that instrumental delivery and the length of the second stage of labor were significantly less in the intervention group [21].

The approaches and opinions of the treatment team regarding the management of the second stage of labor in persistent occiput posterior are different [2, 12, 13]. Management of this position potentially depends on the experience and skill of the operator and the available scientific awareness [8].

According to the survey, most midwives and gynecologists announced that the reason for not performing this technique was the lack of sufficient training and necessary practical skills, as well as not mentioning it in the national protocol.

Most of them were eager to perform this technique If they were trained.

According to the majority of participants, if this technique is a safe and uncomplicated method for the mother and fetus, reduces instrumental delivery and cesarean section, and is introduced as a national protocol, they are willing to learn and perform it.

## Conclusion

According to the results of this study, midwives and gynecologists consider manual rotation in the second stage of labor acceptable; however, they have not received enough training and have not acquired the necessary skills to perform it. Therefore, in order to promote vaginal delivery, reduce maternal and newborn complications, and decrease instrumental and cesarean delivery, adequate training should be done and included in the national protocol.

## Electronic supplementary material

Below is the link to the electronic supplementary material.

Supplementary Material 1

## Data Availability

All data generated or analysed during this study are included in this published article [and its supplementary information files].
